# Characterization and Expression Analysis of MicroRNAs in the Tube Foot of Sea Cucumber *Apostichopus japonicus*

**DOI:** 10.1371/journal.pone.0111820

**Published:** 2014-11-05

**Authors:** Hongdi Wang, Shikai Liu, Jun Cui, Chengze Li, Xuemei Qiu, Yaqing Chang, Zhanjiang Liu, Xiuli Wang

**Affiliations:** 1 Key Laboratory of Mariculture and Stock Enhancement in North China's Sea, Ministry of Agriculture, Dalian Ocean University, Dalian, China; 2 The Fish Molecular Genetics and Biotechnology Laboratory, School of Fisheries, Aquaculture and Aquatic Sciences and Program of Cell and Molecular Biosciences, Aquatic Genomics Unit, Auburn University, Auburn, Alabama, United States of America; Chinese Academy of Fishery Sciences, China

## Abstract

MicroRNAs (miRNAs) are a class of endogenous non-coding small RNA with average length of 22 nucleotides, participating in the post-transcriptional regulation of gene expression. In this study, we report the identification and characterization of miRNAs in the tube foot of sea cucumber (*Apostichopus japonicus*) by next generation sequencing with Illumina HiSeq 2000 platform. Through the bioinformatic analysis, we identified 260 conserved miRNAs and six novel miRNAs from the tube foot small RNA transcriptome. Quantitative realtime PCR (qRT-PCR) was performed to characterize the specific expression in the tube foot. The results indicated that four miRNAs, including miR-29a, miR-29b, miR-2005 and miR-278-3p, were significantly up-regulated in the tube foot. The target genes of the four specifically expressed miRNAs were predicted *in silico* and validated by performing qRT-PCR. Gene ontology (GO) and KEGG pathway analyses with the target genes of these four miRNAs were conducted to further understand the regulatory function in the tube foot. This is the first study to profile the miRNA transcriptome of the tube foot in sea cucumber. This work will provide valuable genomic resources to understand the mechanisms of gene regulation in the tube foot, and will be useful to assist the molecular breeding in sea cucumber.

## Introduction

MicroRNAs (miRNAs) are a class of endogenous non-coding RNA in length of 22 nucleotides (nt) on average. The miRNAs could post-transcriptionally regulate gene expression of growth, development, differentiation and many other biological processes [1]. The regulation is realized through hybridization of the miRNA to the 3′ untranslated region (UTR) of target mRNA, which can be in various ways including translational repression, mRNA cleavage and deadenylation [1], [2].

The processes involved in the miRNA biogenesis have been well demonstrated [3]. Briefly, a primary miRNA (pri-miRNA) is first transcribed from a miRNA gene by the RNA polymerase II or III enzyme in the nucleus. After being cleaved by a complex composed of RNase III enzyme Drosha, the pri-miRNA is transformed into precursor miRNA (pre-miRNA), a short stem-loop structure (∼60–70 nt) [4], [5]. The pre-miRNA is transported across the nuclear membrane and then processed by another RNase enzyme, Dicer, to produce the mature miRNA (∼18–25 nt) in the cytoplasm [6], [7]. The mature miRNA functions by being incorporated into an RNA-induced silencing complex (RISC) to regulate the gene expression post-transcriptionally.

Since the first miRNA was found in *Caenorhabditis. elegans* in 1993 [8], a large number of miRNAs have been identified from numerous species, including plants [9], viruses [10], invertebrates [11]–[14] and vertebrates [15]–[17]. For instance, a total of 24,521 hairpin precursor miRNAs expressing 30,424 mature miRNA products in 206 species are deposited in the public miRNA database, miRBase (http://www.mirbase.org/, Release 20). Among these species, a number of aquatic animals were sequenced to identify the miRNAs in recent years, including several fish species [16], [18]–[22], echinoderms [12], [23]–[25] and others [14].

Sea cucumber, *Apostichopus japonicus* (Echinodermata, Holothuroidea), an echinoderm species, distributes widely along the coasts of China, Japan, Korea and Russia of Northeast Asia [26]. Owing to its great economic value, sea cucumber was massively cultured in the Asia, especially in China [27]. Among the echinoderm species, the miRNAs have been identified in sea urchin (*Strongylocentrotus purpuratus* and *S. Nudus*) [23], [24], sea star (*Patiria miniata*) [25], as well as sea cucumber (*A. japonicus*). Li et al. (2012) identified the miRNAs from haemocytes in coelomic fluid of the *A. japonicus* where they focused on the identification of miRNAs differentially expressed between individuals with skin ulceration syndrome (SUS) and healthy individuals [12]. Du et al. (2013) reported the identification of differentially expressed miRNAs from the intestine of sea cucumber (*A. japonicus*) between aestivation and non-aestivation individuals [28]. To the best of our knowledge, the identification and expression analysis of miRNAs in the tube foot of sea cucumber (*A. japonicus*) have not been reported. The tube foot is an important part of body wall of the sea cucumber, which functions in locomotion, feeding and respiration. However, the molecular mechanisms underlying these biological processes remain largely unknown.

In this study, we report the identification of miRNAs from the tube foot of sea cucumber (*A. japonicus*) by deep sequencing of small RNA transcriptome with the Illumina HiSeq 2000 platform. The main objective of this study is to analyze the transcriptional profiles of the miRNAs and identify miRNAs that are specifically expressed in the tube foot of sea cucumber. This work will provide valuable genomic resources to understand the mechanisms of gene regulation in the tube foot, and will assist in the molecular breeding of sea cucumber.

## Results

### Deep sequencing of small RNA transcriptome

**Figure 1:**
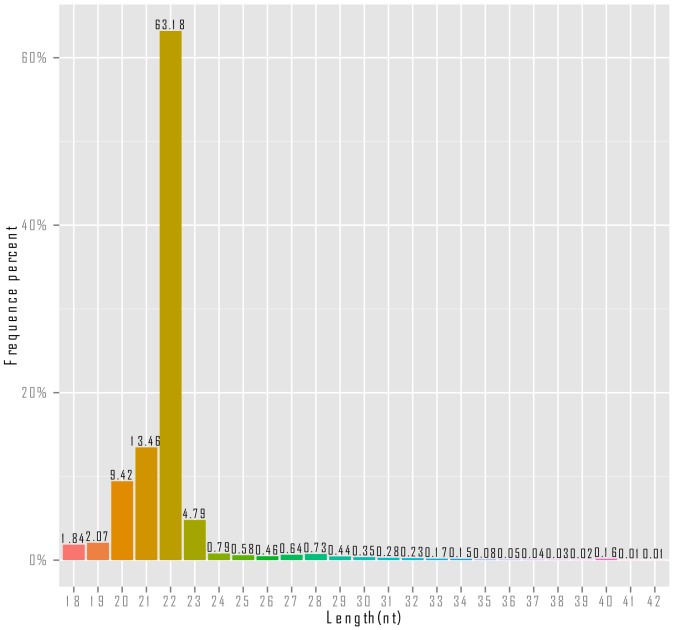
Length distribution of small RNAs identified from the tube foot of sea cucumber (*A. japonicus*).

A total of 8,053,300 raw reads were generated by sequencing the small RNA transcriptome of the tube foot using HiSeq 2000 platform. After trimming, a total of 4,799,733 clean reads were obtained (Table 1). As shown in Figure 1, it's clear that the majority of the small RNAs were in length of 20 nt to 23 nt. The small RNAs with the length of 22 nt were the most abundant (63.18%), followed by the small RNAs with the length of 21 nt (13.6%), 20 nt (9.42%) and 23 nt (4.79%).

**Table 1:** Summary of the small RNA transcriptome sequencing of the *A. japonicus* tube foot.

	Number of sequenced reads	Percentage
***Total reads***	8,053,300	100.0%
***Low quality score***	1,192	0.01%
***5^′^ adapter contaminant***	737	0.01%
***3^′^ adapter null and insert null***	3,232,436	40.14%
***Poly A/T/G/C***	19,202	0.24%
***N%>10%***	0	0%
***Clean reads***	4,799,733	59.60%

The clean reads were then annotated according to their similarities to the non-coding RNAs from public database (Rfam noncoding RNA database). In the cases that some small RNAs were aligned to more than one category, the following priority order was applied: miRNA> rRNA> tRNA> snRNA> snoRNA> novel miRNA. The summary of the identified non-coding RNAs was provided in Table 2. All the reads annotated as tRNA, rRNA, snoRNA and snRNA were removed from further analysis, while the remaining small RNA reads were used for miRNA identification.

**Table 2:** Summary of small RNA analysis.

	Number of sequenced reads	Percentage
***All clean reads***	4,799,733	100%
***miRNA***	3,206,438	66.8%
***rRNA***	38,500	0.80%
***tRNA***	11,948	0.25%
***snRNA***	194	0.00%
***snoRNA***	211	0.00%
***novel miRNA***	8,751	0.17%
***Others***	1,533,691	32.0%

### Identification of miRNAs

To identify the conserved miRNAs, the small RNA reads were searched against all the metazoan miRNAs from miRBase (Release 20.0). A total of 3,206,438 reads were derived from 260 conserved miRNAs. Of the 260 miRNAs, the number of miRNAs with sequenced reads greater than 100 was 67. The detailed information of all the identified miRNAs was provided in Table S1.

**Figure 2:**
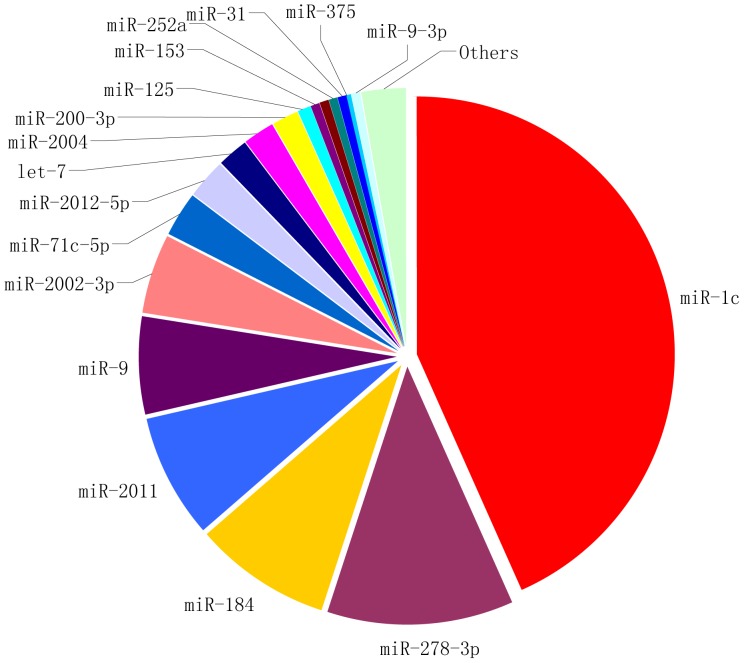
Relative abundance of conserved miRNAs identified from the tube foot of sea cucumber (*A. japonicus*).

Based on the number of sequenced reads, the relative abundance of conserved miRNAs in the tube foot was assessed. As shown in Figure 2, miR-1c was the most abundantly expressed miRNA in the tube foot, followed by miR-278-3p and miR-184.

**Figure 3:**
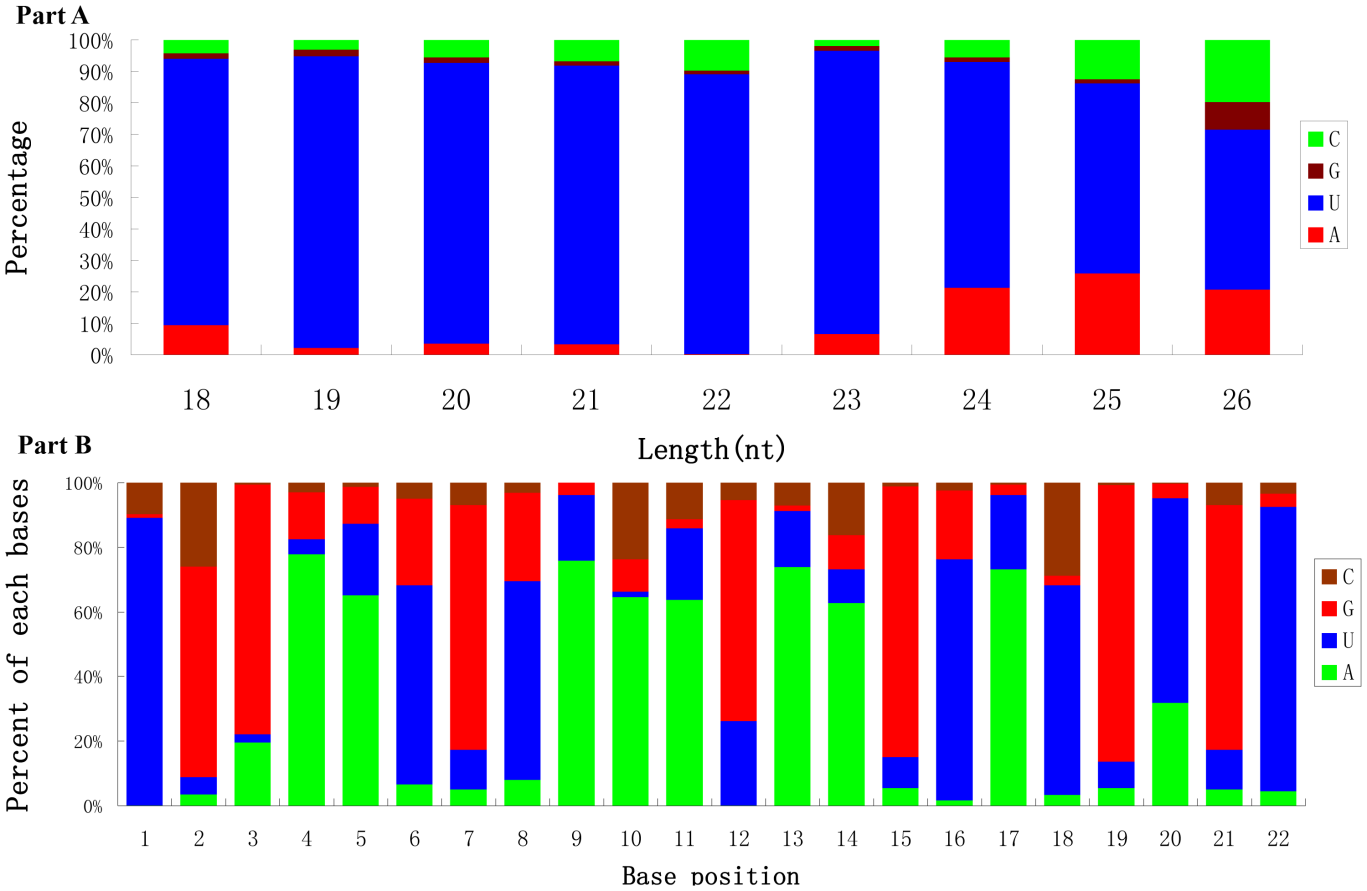
Analyses of the nucleotide bias at the first position of miRNAs and each position of miRNAs with length of 22 nucleotides. A: The nucleotide bias at the first position of miRNAs. B: The nucleotide bias at each position of miRNAs with length of 22 nucleotides.

The nucleotide bias at the first position of the identified miRNAs was analyzed (Figure 3A). The miRNAs showed a significant bias to uracil (U) at the first nucleotide, which is especially true for the miRNAs with lengths of 18 nt to 23 nt. The percentages of the four nucleotides appearing at each position were also analyzed for the miRNAs with the length of 22 nucleotides. As shown in Figure 3B, it's clear that the 2^nd^, 3^rd^, 7^th^ nucleotides are biased to G+C, and the 4^th^, 5^th^, 9^th^, 10^th^ and 11^th^ are significantly biased to A+U.

**Figure 4:**
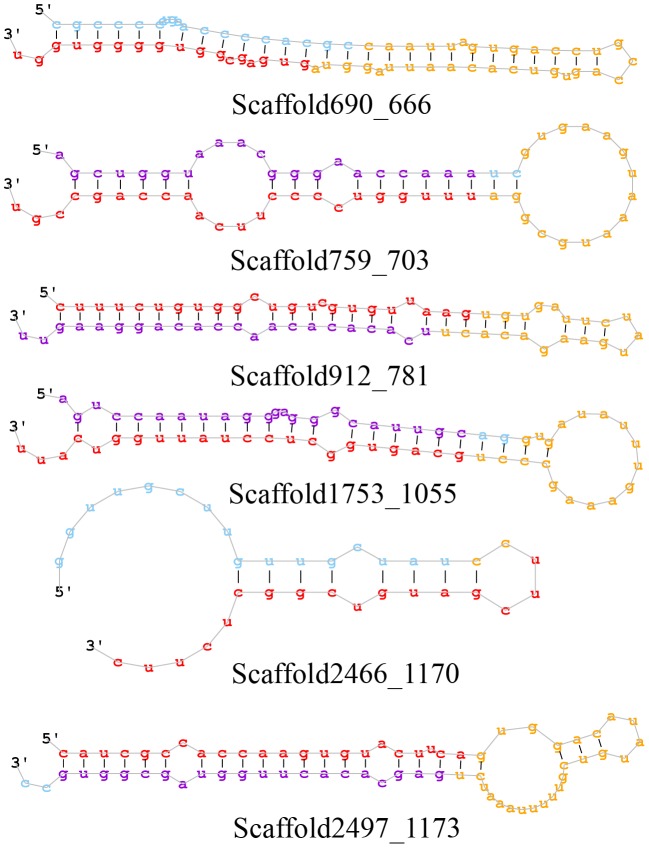
The secondary structure of predicted novel miRNAs.

The remainder of small RNA reads was subject to the prediction for novel miRNAs. A total of six miRNAs were predicted as candidate novel miRNAs. The detailed information of the six novel miRNAs were shown in Table 3, and the corresponding secondary structures were illustrated in Figure 4.

**Table 3:** Novel miRNA candidates identified from tube foot of sea cucumber.

Provisional ID	miRDeep^2^ score	Read counts	miRNA sequences	Consensus precursor sequence
Scaffold2497_1173	3.5e+3	6,984	caucgccaccaaguguacuuca	caucgccaccaaguguacuucaguggacauaugucguuuuuaaaucugagcacacuugguagcggug
Scaffold912_781	6.6e+2	1,292	cuuucuguggcugucguguuaag	cuuucuguggcugucguguuaagugugauucuaugaagacacuucacacacaaccacaggaaguu
Scaffold1753_1055	3.1e+1	58	gcaguggcuccuauuggucauu	aguccaauagggagggcauugcaggugauauuugaaagcccugcaguggcuccuauuggucauu
Scaffold759_703	2.5e+1	47	uuugguccccuucaaccagccgu	agcugguaaacgggaaccaaaucgugaaguaaaugcggauuugguccccuucaaccagccgu
Scaffold690_666	2.2	160	gugagcggugggguggu	cgcccccugaaccccacgccaauuagugaccugccagugucacaauuagguagugagcggugggguggu
Scaffold2466_1170	1.5	210	uucgaugucggcucuuc	gguugcuuguugcuauccuucgaugucggcucuuc

### Prediction and analysis of target genes

**Figure 5:**
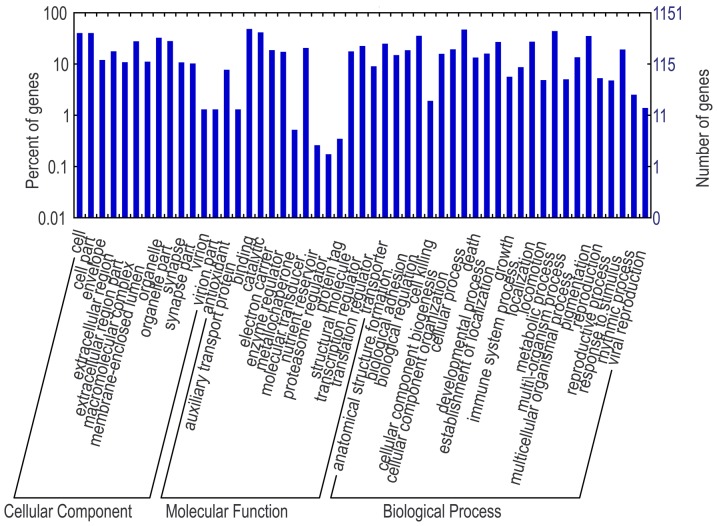
GO terms for predicted target genes.

In total, 16,331 and 15,758 reads were extracted from 3′-UTR and 5′-UTR regions [29]. With these sequences, the putative target genes of identified miRNAs were predicted using the program miRanda-3.3 [30]. The sequences of predicted target genes were listed in Table S2. To understand the functional networks of the target genes, GO analysis was performed. GO analysis showed that these target genes were involved in a large number of physiological processes at the levels of molecular function, cellular component and biological processes. The GO terms were relatively even-distributed in different categories of GO terms with the exception of molecular function where the binding and protein binding terms were dominant (Figure 5).

### qRT-PCR validation

**Figure 6:**
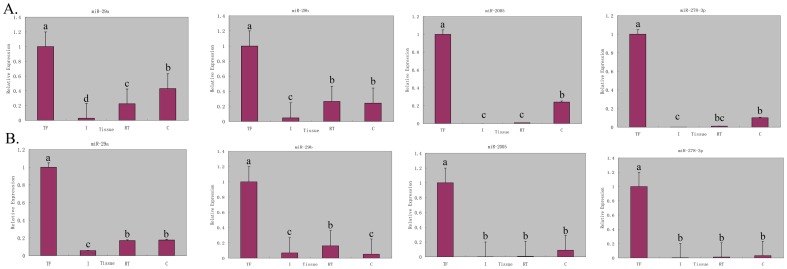
Quantitative realtime PCR analysis of the expression of miR-29a, miR-29b, miR-2005 and miR-278-3p in different tissues of sea cucumber. The tissues are abbreviated as follows: TF, tube foot; I, intestine; RT, respiratory tree, and C, coelomocytes. A: qRT-PCR using *β-actin* as the reference gene. B: qRT-PCR using *Cytb* as the reference gene. Bars are shown as mean ± standard deviation. Bars with different superscripts indicate that they are significantly different from each other (*p*<0.05).

To analyze the tissue-specific expression of the identified miRNAs, 19 miRNAs were selected to perform quantitative real-time PCR (qRT-PCR) analysis in four different tissues, including intestine, respiratory tree, hemocyte and tube feet. As shown in Figure 6, four of the 19 selected miRNAs, i.e. miR-29a, miR-29b, miR-2005 and miR-278-3p, were significantly up-regulated in the tube foot of *A. japonicus*. Therefore, these four miRNAs were considered as the candidates that were specifically expressed in the tube foot.

**Figure 7:**
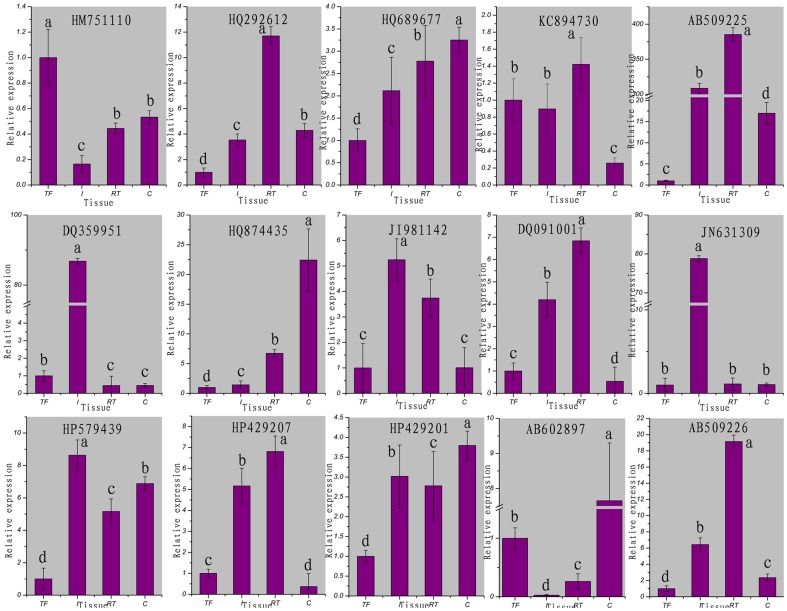
Quantitative realtime PCR analysis of the expression of 15 selected predicted target genes in different tissues of sea cucumber. The tissues are abbreviated as follows: TF, tube foot; I, intestine; RT, respiratory tree, and C, coelomocytes. Bars are shown as mean ± standard deviation. Bars with different superscripts indicate that they are significantly different from each other (*p*<0.05).

To examine the regulatory roles of the miRNAs, we selected 15 target genes of the tube foot specifically expressed miRNAs for expression analysis with qRT-PCR. As shown in Figure 7, eight target genes were significantly repressed in the tube foot comparing to other tissues, i.e. HQ292612, HQ689677, AB509225, HQ874435, HP579439, HP429201, AB602897 and AB509226. Another three genes including JI981142, DQ091001 and HP429207 were also down-regulated in the tube foot, though the down-regulation was also observed in other tissues such as the coelomocytes (Figure 7). These results indicated that the identified miRNAs repressed the expression of their target genes in the tube foot of *A. japonicus*. On the other hand, the identification of tube foot specifically expressed miRNAs and their respective target genes were validated.

### Analysis of target genes of miRNAs specifically expressed in the tube foot

With the four validated miRNAs specifically expressed in the tube foot, we investigated on the pathways that are formed by genes regulated by these miRNAs. All the available nucleotide sequences from NCBI GenBank and previous studies [29] were used for prediction of target genes with the programs PITA [31] and miRanda-3.3 [30]. The genes predicted by both programs were considered as potential target genes. The numbers of predicted target genes for miR-29a, miR-29b, miR-2005 and miR-278-3p were 560, 536, 496 and 272, respectively.

**Figure 8:**
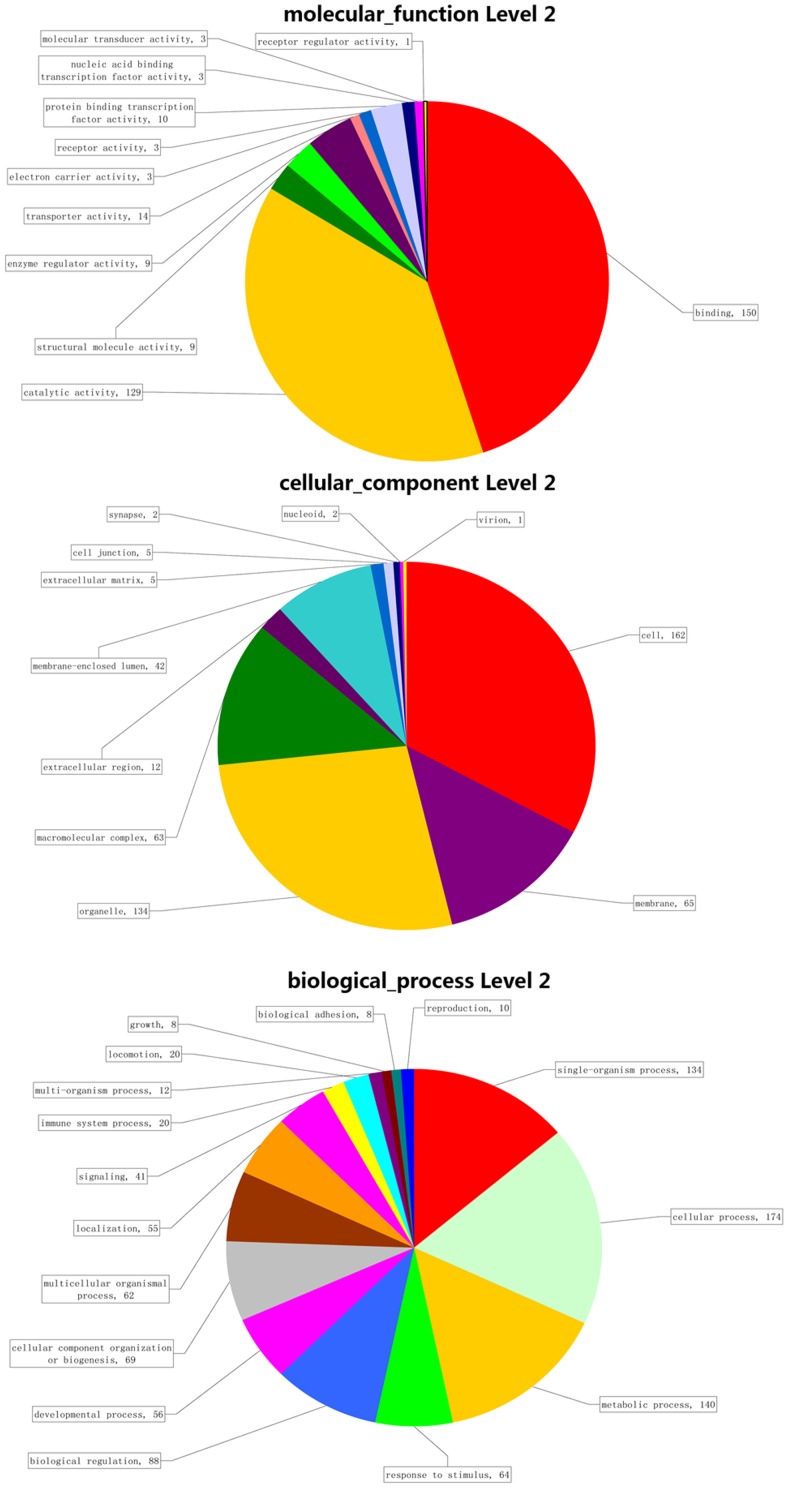
GO analysis for predicted target genes of miR-29a at level 2.

For each of the tube foot specifically expressed miRNAs, the distribution of the target genes in different GO categories at level 2 was shown in Figure 8, and in Figure S1, Figure S2 and Figure S3, respectively. Take miR-29a as an instance, 413 (73.75%) of the 560 target genes were annotated and assigned with one or more GO terms of biological process (202 genes), molecular function (213 genes) and cellular component (176 target genes). The GO term distributions of target genes of the four miRNAs were in a similar pattern. The highly represented GO terms were metabolic process and cellular process for biological process, and binding and catalytic activity for molecular function, while major categories for cellular components were cell part and cell terms.

**Figure 9:**
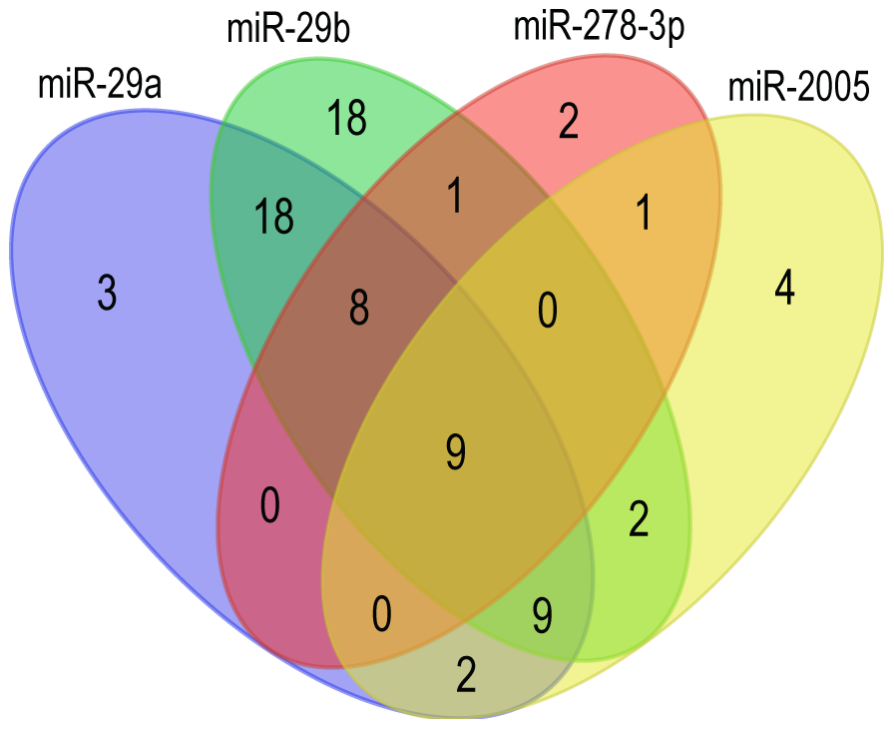
Venn diagram of the identified KEGG pathways.

The KEGG pathway analysis suggested that a total of 49, 65, 27 and 21 pathways were mapped with the target genes of miR-29a, miR-29b, miR-2005 and miR-278-3p, respectively. By comparing the pathways formed by target genes of the four tube foot specifically expressed miRNAs, the shared pathways were obtained (Figure 9). Of which, nine pathways were shared by all the four tube foot specific miRNAs, eight pathways were shared by miR-29a, miR-29b and miR-278-3p, while nine pathways were shared by miR-29a, miR-29b and miR-2005. A number of 18 pathways were shared by miR-29a and miR-29b. The detailed information of these shared pathways was provided in Table S3. Several shared pathways involved in metabolisms of amino acid, fatty acid, glycosaminoglycan, and minor element were of interest and were discussed below.

## Discussion

Many studies have reported that the tube foot is a multifunctional organ for sea cucumber, including movement, adsorption, respiration, sensation and immune response [32]. Meanwhile, the tube foot is an important part of body wall which is the main product for sea cucumber food. Providing new information of the tube foot of sea cucumber at the molecular level, including miRNAs at transcriptional level, is essential to understand the molecular mechanisms underlying biological processes in the tube foot of sea cucumber. The miRNA transcriptome sequencing has been conducted in sea cucumber using the high throughput sequencing recently. Li et al. (2012) and Du et al. (2013) reported the analysis of the miRNA transcription in the haemocyte and intestine respectively [12], [28]. In those two studies, the differentially expressed miRNAs among different physiological conditions were mainly investigated. The tissue-specific expression pattern of miRNAs was not analyzed because only single tissue was used in each of their studies. To the best of our knowledge, analysis of miRNA transcriptome in the tube foot of sea cucumber has not been conducted. In this study, we performed the deep sequencing of the tube foot of *A. japonicas* to characterize the miRNA transcriptome. Furthermore, we were able to identify the predominantly expressed miRNAs in the tube foot by taking advantage of the published data generated from the haemocyte and intestine [12], [28].

In this study, a total of 4,799,733 clean reads were generated, which were fewer than that generated by Li et al. and Du et al. [12], [28]. One of the reasons is that total RNA in the tube foot is much less abundant than that in the haemocyte and intestine [28]. According to the length distribution of small RNAs, over 84.4% of the sequenced read had the length of 21–23 nt, which is the typical size range of miRNA. The length distribution was consistent with that in the studies of Li et al. and Du et al., and in other echinoderms [23]–[25], suggesting the sequencing was as good as in previous studies.

In this study, we have found 260 conserved miRNAs and six novel miRNAs. The variable expression of these conserved miRNAs was observed. Three of the abundantly expressed miRNAs were miR-1, miR-278-3p and miR-184, which is different from the observation in Li et al [10] and Du et al [26]. Li et al. found that miR-10 was the most abundantly expressed miRNA and Du et al. found that miR-10a and miR-10a-5p were two of the most abundantly expressed miRNAs. The miRNA expression varied from different tissues, development stages and physiological conditions. In this work, we sequenced the tube foot of healthy sea cucumbers, while in studies of Li et al. and Du et al., the haemocytes in coelomic fluid and intestine were used, respectively. Moreover, individuals with skin ulceration syndrome and in aestivation status were examined in their studies [12], [28].

Analysis of the distribution of the four nucleotides at each position along the length of miRNAs revealed that uracil predominated at several other positions including the 1^st^ nucleotide, and the 9^th^ nucleotide where both adenosine and uracil are predominant. This is consistent with the observation based on the systematic analysis of thousands of metazoan miRNAs [28]. In canonical cases, the 2^nd^ to the 8^th^ nucleotides of miRNAs, named as “seed region”, are perfectly complementary with their target sites. Therefore, the “seed region” is known to play a critical role in targeting mature miRNAs to mRNAs for regulation [33]. The strong bias of uracil at the 1^st^ and 9^th^ nucleotides that immediately flank the edges of the “seed region” indicated that they may play important roles in miRNA regulatory mechanisms.

We identified four miRNAs, miR-29a, miR-29b, miR-2005 and miR-278-3p, which were predominantly expressed in the tube foot. The results were well supported by the qRT-PCR analysis. The target genes of these four miRNAs were predicted *in silico* and the expression profiles were examined by performing qRT-PCR. Eleven of the 15 selected target genes were down-regulated in the tube foot, while the other four target genes were not. As for the reasons, one miRNA could target multiple genes [1], [5], and vice versa, one gene could be regulated by multiple miRNAs. The target genes that we selected to perform qRT-PCR could be mainly regulated by other miRNA(s) that were not identified in the present work. In addition, the post-transcriptional regulation is a complicated process which is not only regulated by miRNAs.

GO analysis and KEGG analysis were performed for target genes predicted by these four tube foot predominant miRNAs. GO analysis results showed that the predicted target genes participated in numerous biological processes, suggesting that the four miRNAs could play important roles in regulation of extensive biological processes. KEGG pathway analysis showed that the target genes were extensively involved in a number of pathways. The shared pathways among these miRNAs were probably most important to investigate on the regulatory function of these four predominant miRNAs in the tube foot.

Homologous to body wall during embryonic development, the tube foot is a part of body wall which is the main product of sea cucumber [34]. Information of the nutriment metabolism in the tube foot will be valuable for the analysis of the nutriment metabolism in body wall, such as metabolisms of amino acid, fatty acid, glycosaminoglycan, and minor element.

Twelve pathways which were shared more than two miRNAs were related to amino acid mechanism, biosynthesis and degradation. Because the composition and content of amino acid in sea cucumbers were related to the flavor and nutritional value, the metabolism of amino acid in sea cucumber is a hot topic to many researchers and aquaculture breeders [35], [36]. Further studies on the pathways related to amino acid metabolisms should warrant the evidence to explain concentration and different content of various amino acids.

The glycosaminoglycan degradation pathway composed of genes regulated by miR-29a and miR-29b was also observed. Glycosaminoglycan, rich in the body wall of sea cucumber, is an important nutrition component and is of great medical value [37], [38]. It's speculated that the accumulation of the glycosaminoglycan in the tube foot and body wall of sea cucumber is related to the inhibition of glycosaminoglycan degradation pathway by these two miRNAs.

The selenocompound metabolism pathways potentially regulated by miR-29a and miR-29b were observed in the KEGG pathway analysis. This indicated that these tube foot specifically expressed miRNAs may affect the metabolism of selenium in tube foot of sea cucumber. The trace mineral, selenium, plays an important and unique role in many biological processes in human. Selenium has been demonstrated to play roles in antioxidant defense, redox state regulation, the immune response and cancer prevention [39], [40]. Previous studies reported that the selenium enriched in sea cucumber is one of the most valuable nutritional components to human health [41], [42]. Further investigation is required to explore the molecular mechanism for the regulation of selenocompound metabolism by miR-29a and miR-29b.

Fatty acid degradation pathway was also found in the result of KEGG pathway analysis. This indicated that the degradation of fatty acid may be negatively regulated by the tube foot predominant miRNAs, resulting in the enrichment of fatty acid in tube foot of sea cucumber. Fatty acid, especially unsaturated fatty acid, is essential for the human diet [43], [44]. Of the 19 fatty acids found in the body wall of sea cucumber, 15 are unsaturated fatty acid, 10 are polyunsaturated fatty acid [35]. Further studies on the fatty acid degradation pathway analysis warrant the dissection of the molecular mechanism involved in the fatty acid metabolism in the body wall of sea cucumber.

## Materials and Methods

### Ethics statement

All procedures involving the handling and treatment of sea cucumber during this study were approved by the Animal Care and Use committee of Key Laboratory of Mariculture & Stock Enhancement in North China's Sea at Dalian Ocean University.

### Samples and RNA extraction

Samples were collected from 16 sea cucumbers (*A. japonicus*) (average weight of 150 g) provided by the Key Laboratory of Mariculture in North China (Dalian, Liaoning). Four tissues, including the intestine, respiratory tree, hemocyte and tube foot, were dissected from each individual and stored in RNAlater (Ambion). The samples were kept at room temperature for 24 hours and then transferred to −80°C freezer until RNA extraction.

Total RNA of was isolated using Trizol reagent (Takara, Dalian) according to the manufacturer's instruction. The RNA quantity and integrity were assessed using the RNA Nano 6000 Assay Kit of the Agilent Bioanalyzer 2100 system (Agilent Technologies, CA, USA), ensuring high quality RNA for the construction of miRNA library.

### Next generation sequencing and data analyses

Sequencing libraries were constructed using NEBNext Multiplex Small RNA Library Prep Set for Illumina (NEB, USA.) following manufacturer's protocol. Briefly, the small RNA was ligated with 3′ and 5′ adapters and purified on 8% polyacrylamide gel (100 V, 80 min). PCR production of small RNA with adaptors with the sizes ranging from 140 bp to 160 bp were collected and dissolved in 8 µL elution buffer. The quality of library was assessed on the Agilent Bioanalyzer 2100 system using DNA High Sensitivity Chips. The clustering of the index-coded samples was performed on a cBot Cluster Generation System using TruSeq SR Cluster Kit v3-cBot-HS (Illumia) according to the manufacturer's instructions. After cluster generation, sequencing was performed on an Illumina HiSeq 2000 platform.

Raw sequence data was exported in fastq format. The raw sequence data was trimmed to remove low quality reads including reads with low sequencing quality scores, reads less than 18 nt in length, reads with 5′ adapters but lost 3′ adapters, and reads with low complexity sequences and simple repeats analyzed by RepeatMasker [45]. The clean reads with length ranging from 18 nt to 40 nt were used for further analyses.

The all clean reads were searched against Rfam (http://rfam.sanger.ac.uk/) database for annotation. All the reads annotated as tRNA, rRNA, snoRNA and snRNA were discarded for further analysis. Considering that there was no published genome information of sea cucumber available, the remainder small RNA reads was compared to all metazoan species known miRNA of miRBase 20.0 (http://www.mirbase.org/) to identify conserved miRNAs. Only the perfect matches were considered as conserved miRNAs. Reads that were not aligned to the miRBase database were used to predict novel miRNAs. The mirdeep2 [46] software were used for the novel miRNAs prediction. Only small RNAs met the criteria as follows were considered as novel miRNAs: 1) forming stem-loop secondary structure, 2) possessing Dicer enzyme site and 3) having the minimum free energy.

The high through sequencing dataset of small RNA transcriptome of A. japonicus tube foot has been deposited to NCBI sequence read archive (SRA) database with the accession number of SRA173398.

### Target gene prediction and analyses

As there was no published genome information of sea cucumber, we tried to extract the 3′-UTR and 5′-UTR from the sea cucumber transcriptome that has been published by Du et al. [29]. The sequences of transcroptome containing the extracted 3′-UTR and 5′-UTR were considered as candidate gene databases for target gene prediction. The MiRanda-3.3 [30] software was used to predict the target genes of identified miRNAs. The predicted target genes were aligned by blastx, then the gene ontology (GO) analysis were performed for the target genes by DAVID (david.abcc.ncifcrf.gov) [47].

### Identification of the miRNAs specifically expressed in the tube foot

To identify the miRNAs specifically expressed in the tube foot, the differential expression profiles of miRNAs were achieved by comparison with the data of health sea cucumber haemocyte from Li et al. [12], and intestine from Chen et al. [28]. The differential expression of miRNAs among the tube foot, haemocyte, and intestine was estimated using the formula: 
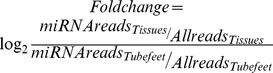


Where *miRNAreads_Tissues_* indicates the number of reads for each of the miRNAs identified in the haemocyte or intestine; *Allreads_Tissues_* indicates the number of all reads for identified miRNAs in the haemocyte or intestine; *miRNAreads_Tubefeet_* indicates the number of reads for each of the miRNAs identified in the tube foot; *Allreads_Tubefeet_* indicates the number of reads for all identified miRNAs in the tube foot. Significant candidates were determined as the miRNAs that had *Foldchange* values>2 or <−2 when compared to both haemocyte and intestine. Followed by the differential expression analysis, 19 candidate miRNAs were selected to perform qRT-PCR for validation.

All nucleotide sequences of *A. japonicus* from GenBank (NCBI) and assembled sequences of sea cucumber transcripts published in Du et al. [29] were downloaded as database to predict the target genes of the four miRNAs specifically expressed in the tube foot using the software miRanda-3.3 [30] and PITA [31]. Sequences predicted by both programs were considered as potential target genes. The parameters of miRanda-3.3 were “S> = 140”, “ΔG< = −17 kcal/mol” and “–strict”, while the default parameters were used in the PITA. Target gene sequences of each miRNA were extracted to perform GO analysis and KEGG pathway analysis using Blast2GO software (www.blast2go.com).

### qRT-PCR validation

qRT-PCR was performed for 19 miRNAs and 15 predicted target genes to validate the results. Stem-loop RT-PCR primers of miRNAs were designed as mentioned in the study of Chen et al. [48]. Primers of target genes were designed following the manufacturer's instruction of SYBR Premix Ex TaqTM II kit (Takara, Dalian). *Cytb* and *β-actin* both were used as reference control genes for miRNAs and *β-actin* was used as reference control genes for mRNA qRT-PCR, respectively. The information of all primers was provided in Table S4. The qRT-PCR was run on ABI 7500 platform and replicated in three pools. The reaction system and progress parameters were set up as in a previous study [49]. The qRT-PCR data was analyzed using 2^-ΔΔCt^ method [50].

## Supporting Information

Figure S1 **GO analysis result at level 2 for predicted target genes of miR-29b.**
(TIF)

Figure S2 **GO analysis result at level 2 for predicted target genes of miR-2005.**
(TIF)

Figure S3 **GO analysis result at level 2 for predicted target genes of miR-278-3p.**
(TIF)

Table S1 **Conserved miRNAs identified from sea cucumber (** ***A. japonicus*** **) tube foot.**
(XLS)

Table S2 **Prediction of miRNA target genes based on the transcriptome of sea cucumber (** ***A. japonicus*** **).**
(FASTA)

Table S3 **Detailed pathways of miRNA target genes identified in the tube foot.**
(DOC)

Table S4 **Primers used in stem loop RT-PCR and qRT-PCR.**
(XLS)
