# Supplementary figures and images for: Characterization and Expression Analysis of MicroRNAs in the Tube Foot of Sea Cucumber Apostichopus japonicus
Source: PLoS One. 2014 Nov 5;9(11):e111820. doi: 10.1371/journal.pone.0111820 (PMC4221132; doi:10.1371/journal.pone.0111820)

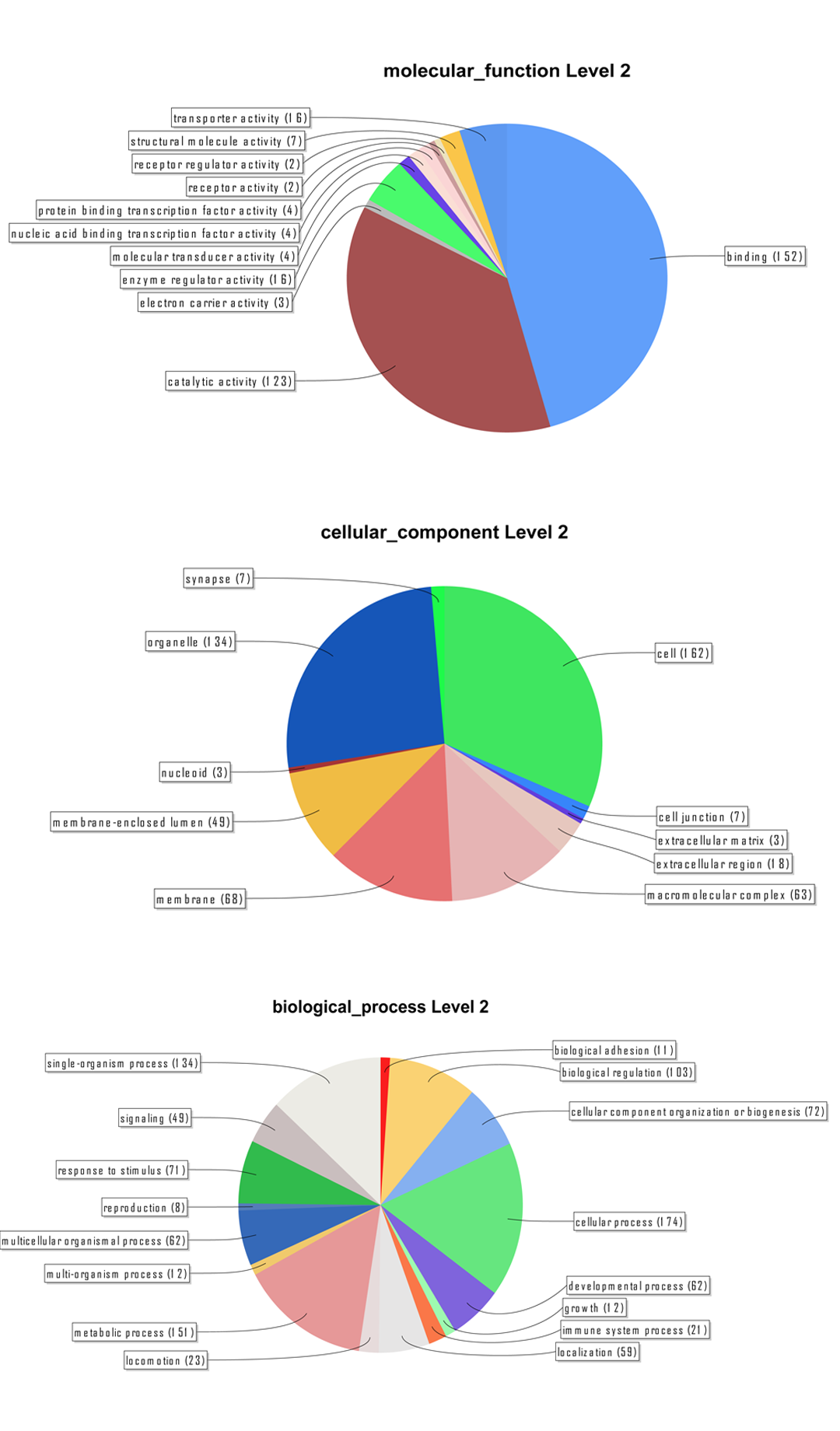

Supplement: Figure S1 — GO analysis result at level 2 for predicted target genes of miR-29b. (TIF) [file pone.0111820.s001.tif]

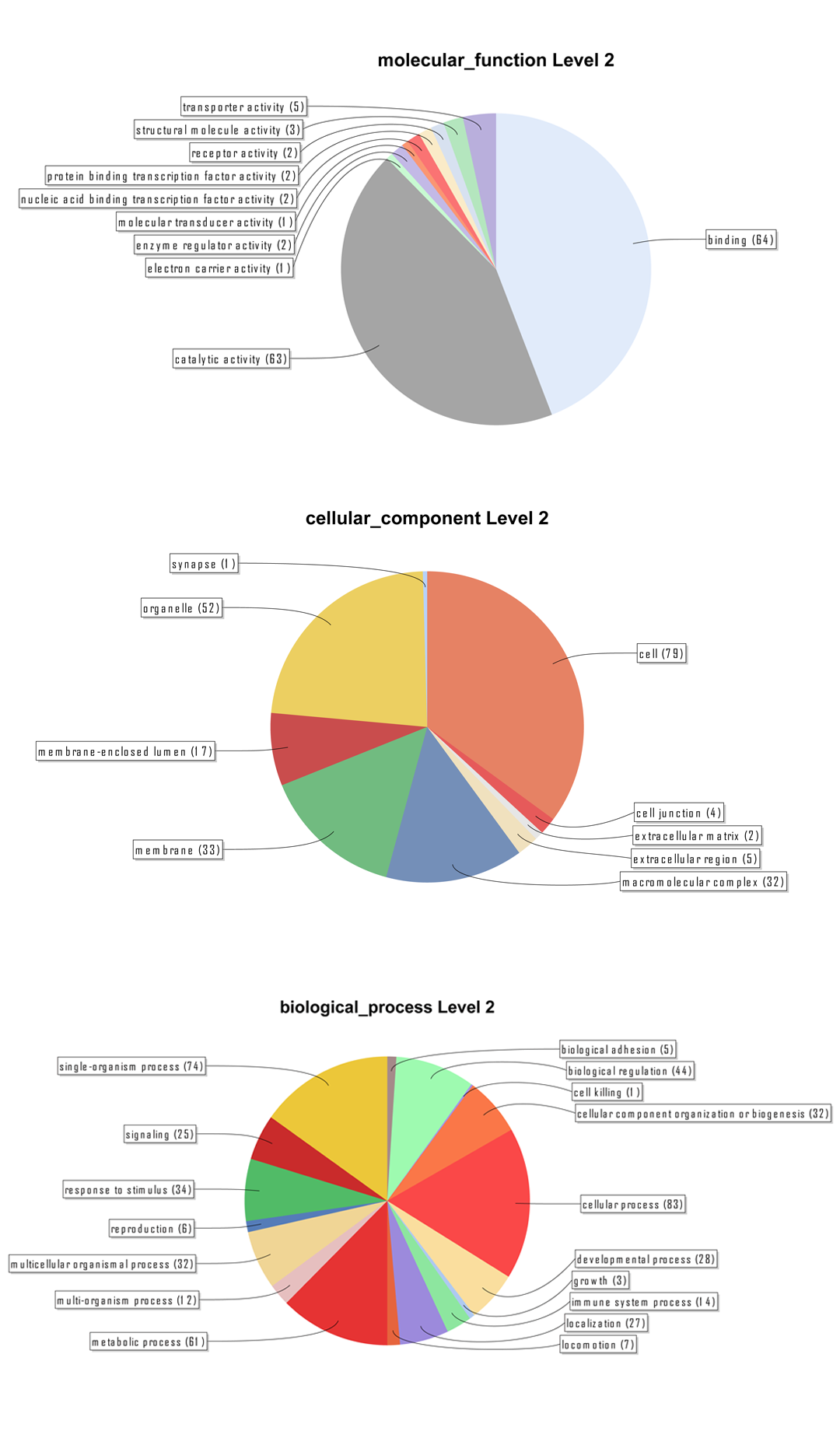

Supplement: Figure S2 — GO analysis result at level 2 for predicted target genes of miR-2005. (TIF) [file pone.0111820.s002.tif]

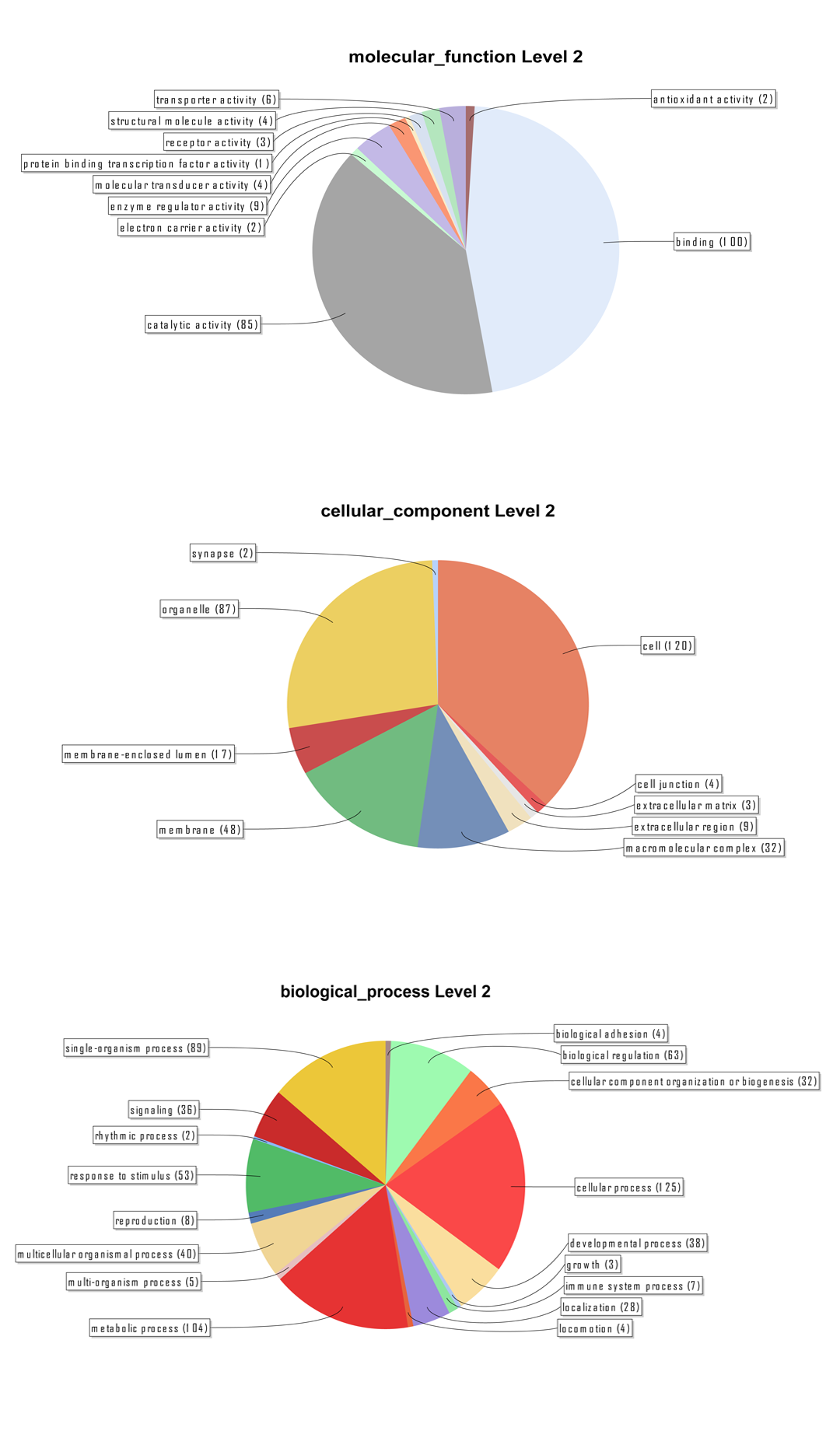

Supplement: Figure S3 — GO analysis result at level 2 for predicted target genes of miR-278-3p. (TIF) [file pone.0111820.s003.tif]
