# Supplementary material for: Characterization and Expression Analysis of MicroRNAs in the Tube Foot of Sea Cucumber Apostichopus japonicus
Source: PLoS One. 2014 Nov 5;9(11):e111820. doi: 10.1371/journal.pone.0111820 (PMC4221132; doi:10.1371/journal.pone.0111820)
Supplement: Table S3 — Detailed pathways of miRNA target genes identified in the tube foot. (DOC) [file pone.0111820.s006.doc]

**Table S3.** Detailed pathways of miRNA target genes identified in the tube foot.

| **miRNAs** | **Shared Pathways Elements** |
| --- | --- |
| miR-2005  miR-278-3p  miR-29a  miR-29b | Glyoxylate and dicarboxylate metabolism |
|  | Citrate cycle (TCA cycle) |
|  | Sulfur metabolism |
|  | Carbon fixation pathways in prokaryotes |
|  | Pentose phosphate pathway |
|  | Amino sugar and nucleotide sugar metabolism |
|  | Aminoacyl-tRNA biosynthesis |
|  | Purine metabolism |
|  | Sphingolipid metabolism |
| miR-278-3p  miR-29a  miR-29b | Starch and sucrose metabolism |
|  | Galactose metabolism |
|  | Lysine degradation |
|  | Arginine and proline metabolism |
|  | Streptomycin biosynthesis |
|  | C5-Branched dibasic acid metabolism |
|  | Propanoate metabolism |
|  | T cell receptor signaling pathway |
| miR-2005  miR-29a  miR-29b | Pyruvate metabolism |
|  | Pyrimidine metabolism |
|  | Methane metabolism |
|  | Tyrosine metabolism |
|  | Alanine, aspartate and glutamate metabolism |
|  | Glycolysis / Gluconeogenesis |
|  | Glutathione metabolism |
|  | Styrene degradation |
|  | Carbon fixation in photosynthetic organisms |
| miR-29a  miR-29b | Novobiocin biosynthesis |
|  | Selenocompound metabolism |
|  | Isoquinoline alkaloid biosynthesis |
|  | Fatty acid degradation |
|  | Tropane, piperidine and pyridine alkaloid biosynthesis |
|  | Histidine metabolism |
|  | Tryptophan metabolism |
|  | Cysteine and methionine metabolism |
|  | Thiamine metabolism |
|  | Phenylalanine, tyrosine and tryptophan biosynthesis |
|  | Glycosaminoglycan degradation |
|  | Oxidative phosphorylation |
|  | Phenylalanine metabolism |
|  | Glycosphingolipid biosynthesis - ganglio series |
|  | Ubiquinone and other terpenoid-quinone biosynthesis |
|  | Lysine biosynthesis |
|  | Valine, leucine and isoleucine degradation |
|  | Butanoate metabolism |
| miR-2005  miR-29a | N-Glycan biosynthesis |
|  | Glycine, serine and threonine metabolism |
| miR-278-3p  miR-29b | Aminobenzoate degradation |
| miR-2005  miR-29b | One carbon pool by folate |
|  | mTOR signaling pathway |
| miR-2005  miR-278-3p | Fructose and mannose metabolism |
| miR-29a | Nicotinate and nicotinamide metabolism |
|  | Fatty acid biosynthesis |
|  | Other types of O-glycan biosynthesis |
| miR-29b | Inositol phosphate metabolism |
|  | Cutin, suberine and wax biosynthesis |
|  | Biosynthesis of unsaturated fatty acids |
|  | Geraniol degradation |
|  | Retinol metabolism |
|  | alpha-Linolenic acid metabolism |
|  | Glycerolipid metabolism |
|  | beta-Alanine metabolism |
|  | Other glycan degradation |
|  | Steroid hormone biosynthesis |
|  | PI3K-Akt signaling pathway |
|  | Primary bile acid biosynthesis |
|  | Caprolactam degradation |
|  | Toluene degradation |
|  | Limonene and pinene degradation |
|  | Phosphatidylinositol signaling system |
|  | Fatty acid elongation |
|  | Ethylbenzene degradation |
| miR-278-3p | Mucin type O-Glycan biosynthesis |
|  | Folate biosynthesis |
| miR-2005 | Various types of N-glycan biosynthesis |
|  | Glycerophospholipid metabolism |
|  | Ether lipid metabolism |
|  | Cyanoamino acid metabolism |

Note: The pathways elements presented in the “Shared Pathways Elements” column means that each pathway of which was regulated by miRNAs presented in the “miRNAs” column.
